# Analysis of association between circulating miR-122 and histopathological features of nonalcoholic fatty liver disease in patients free of hepatocellular carcinoma

**DOI:** 10.1186/s12876-016-0557-6

**Published:** 2016-12-12

**Authors:** Norio Akuta, Yusuke Kawamura, Fumitaka Suzuki, Satoshi Saitoh, Yasuji Arase, Shunichiro Fujiyama, Hitomi Sezaki, Tetsuya Hosaka, Masahiro Kobayashi, Yoshiyuki Suzuki, Mariko Kobayashi, Kenji Ikeda, Hiromitsu Kumada

**Affiliations:** 1 Department of Hepatology, Toranomon Hospital and Okinaka Memorial Institute for Medical Research, 2-2-2 Toranomon, Minato-ku, Tokyo, 105-0001 Japan; 2 Liver Research Laboratory, Toranomon Hospital, Tokyo, Japan

**Keywords:** Nonalcoholic fatty liver disease, Nonalcoholic steatohepatitis, Serial liver biopsy, Longitudinal observation, microRNA-122, Circulating

## Abstract

**Background:**

The association between circulating microRNA-122 (miR-122) and histopathological features of nonalcoholic fatty liver disease (NAFLD) remains unclear.

**Methods:**

The association of serum miR-122 levels with histopathological features of NAFLD (steatosis, ballooning, lobular inflammation, and stage, as histological components of nonalcoholic steatohepatitis) was examined in serial liver biopsies from 36 hepatocellular carcinoma (HCC)-free Japanese patients with histopathologically-proven NAFLD. The median interval between first and second liver biopsies was 4.6 years.

**Results:**

In patients who showed improvement of histopathological scores (steatosis, ballooning, and stage), serum miR-122 levels were significantly lower at second biopsy than first biopsy. In patients who showed no improvement, the changes at second biopsy were not different from those at first biopsy. There were significant and strong associations between serum miR-122 ratio (ratio of level at second biopsy to that at first biopsy) and changes in histopathological scores (of steatosis, lobular inflammation, and stage). There were also significant and strong associations between serum miR-122 ratio and changes in other clinical parameters, including aspartate aminotransferase and alanine aminotransferase.

**Conclusions:**

Longitudinal examination of serial liver biopsies showed the association of serum miR-122 with histopathological features of HCC-free NAFLD patients.

**Electronic supplementary material:**

The online version of this article (doi:10.1186/s12876-016-0557-6) contains supplementary material, which is available to authorized users.

## Background

Non-alcoholic fatty liver disease (NAFLD) is currently the most common liver disease worldwide across different ethnicities [1–7], and associated with serious healthcare issues. NAFLD includes a wide spectrum of liver pathologies ranging from non-alcoholic fatty liver, which is usually benign, to non-alcoholic steatohepatitis (NASH), which may lead to liver cirrhosis, hepatocellular carcinoma (HCC), and liver failure without excessive alcohol intake [8]. Treatment with vitamin E and Farnesoid X nuclear receptor ligand obeticholic acid is reported to improve the histological features of NAFLD [9, 10].

NASH can only be diagnosed by the presence of histopathological components, such as steatosis, lobular inflammation, ballooning, and fibrosis. Hence, there is a need for non-invasive surrogate markers of histopathological features. The severity and progression of NAFLD is influenced by various factors, including environmental factors, and genetic and epigenetic variations [11–14]. Recent studies have explored the utility of circulating microRNA for assessment of NAFLD. Several reports indicated that serum levels of several microRNAs were increased in patients with NAFLD, and that serum microRNA-122 (miR-122) levels correlated with histopathological disease severity [15–18]. Based on serial follow-up of rats with experimentally-induced NAFLD, Yamada et al. [19] concluded that serum miR-122 level was indeed useful for the assessment of early NAFLD and could be superior to traditional clinical markers that are often used to monitor liver diseases. Takaki et al. [20] examined HCC tissues of mice with experimentally-induced NASH and concluded that silencing of miR-122 is an early event during hepatocarcinogenesis from NASH, and that miR-122 is potentially suitable for evaluation of the risk of HCC in patients with NASH. In a cross-sectional study based on large number of patients with histopathologically-confirmed NAFLD, we recently identified the absence of HCC and/or histopathological severity of NASH as independent predictors of high serum levels of miR-122. In another study, we followed three HCC patients with histopathologically-confirmed NAFLD and showed a decline in serum miR-122 levels before the progression of fibrosis stage [21]. However, there is no information at present on the longitudinal effect of serum miR-122 on histopathological features of NAFLD.

The aim of this single-center retrospective cohort study was to determine the long-term effects of serum miR-122 on histopathological features of NAFLD in patients free of HCC.

## Methods

### Patients

A total of 321 Japanese patients were diagnosed with NAFLD based on histopathological examination of liver biopsies between 1980 and 2016 at Toranomon Hospital. Of these, 39 patients underwent at least two liver biopsies and were evaluated in detail clinically over time. The need for repeated liver biopsies was determined by the attending physician. Of the 39 patients, 36 did not develop HCC during the period from the first to the second biopsy (median: 4.6 years, range: 0.5-19.0 years). The association between serum miR-122 and histopathological features of NAFLD in these 36 patients free of HCC was evaluated longitudinally.

NAFLD was diagnosed based on liver histopathological findings of steatosis in ≥5% of hepatocytes and the exclusion of other liver diseases (such as primary biliary cirrhosis, autoimmune hepatitis, drug induced liver disease, viral hepatitis, hemochromatosis, biliary obstruction, α-1-antitrypsin deficiency-associated liver disease, and Wilson disease). None of the 36 patients consumed more than 20 g/day alcohol.

The study protocol was in compliance with the Good Clinical Practice Guidelines and the 1975 Declaration of Helsinki, and was approved by the institutional review board of Toranomon Hospital. All patients provided written informed consent at the time of liver histopathological diagnosis.

### Liver histopathology

Liver specimens were obtained using a 14-gauge modified Vim Silverman needle (Tohoku University style, Kakinuma Factory, Tokyo, Japan), a 16-gauge core tissue biopsy needle (Bard Peripheral Vascular Inc., Tempe, AZ) or surgical resection. The biopsy tissue sample was fixed in 10% formalin, and sections were stained with hematoxylin-eosin, Masson trichrome, silver impregnation, and periodic acid-Schiff after diastase digestion. The specimens were evaluated by four pathologists (K.K., F.K., T.F., and T.F.) who were blinded to the clinical findings. An adequate liver biopsy sample was defined as a specimen more than 1.5 cm long tissue strip and/or containing more than 11 portal tracts. Specimens with steatosis of <5%, 5–33%, 34–66%, and >66% were scored as steatosis grade 0, 1, 2, and 3, respectively. Lobular inflammation of no foci, <2 foci, 2-4 foci, and >4 foci per 200× field was scored as 0, 1, 2, and 3, respectively. Hepatocyte ballooning of none, few cells, and many cells was scored as 0, 1, and 2, respectively. NAFLD activity score represented the sum of steatosis, lobular inflammation, and hepatocyte ballooning scores (range, 0–8 points; 5–8 points as definition of NASH). Fibrosis stage of none, zone 3 perisinusoidal fibrosis (stage 1), zone 3 perisinusoidal fibrosis with portal fibrosis (stage 2), zone 3 perisinusoidal fibrosis and portal fibrosis with bridging fibrosis (stage 3), and cirrhosis (stage 4) was scored as 0, 1, 2, 3, and 4, respectively [22, 23]. Patients were also classified into four categories by histopathology according to the classification of Matteoni et al. [24] as follows; type 1: fatty liver alone, type 2: fat accumulation and lobular inflammation, type 3: fat accumulation and ballooning degeneration, type 4: fat accumulation, ballooning degeneration, and either Mallory-Denk body or fibrosis (type 3 or 4 as definition of NASH).

A decrease of one point or more in the histopathological score at the time of second biopsy (relative to the first biopsy) was classified as "improvement", no change as “no change”, while an increase of one point or more was termed “progression”. Changes in histopathological score (Δchange) represented the score at the second biopsy minus that at the first biopsy.

### Clinical parameters

Table 1 summarizes the clinical features of 36 patients of NAFLD free of HCC recorded at the time of first and second biopsies. The normal ranges of aspartate aminotransferase (AST) and alanine aminotransferase (ALT) at our hospital are 13-33 IU/l and 8-42 IU/l for males and 6-27 IU/l for females, respectively. Obesity was defined as body mass index (BMI) of more than 25.0 kg/m^2^. Non high-density lipoprotein cholesterol was defined as total cholesterol minus high-density lipoprotein cholesterol. Changes in clinical parameters or laboratory tests represented the value at second biopsy minus that at first biopsy.

**Table 1:** Clinical characteristics at the time of the first and second liver biopsies, of 36 patients with NAFLD free of hepatocellular carcinoma

	First biopsy	Second biopsy
Demographic data		
Number of patients	36	36
Gender (Male/Female)	20/16	20/16
Histological findings		
Steatosis (0/1/2/3)	0/8/20/8	1/15/15/5
Lobular inflammation (0/1/2/3)	4/14/13/5	2/20/14/0
Ballooning (0/1/2)	2/21/13	4/23/9
Stage (0/1/2/3/4)	5/13/6/11/1	2/16/5/12/1
Matteoni classification (type 1/2/3/4)	1/1/3/31	0/1/1/33
NAFLD activity score (≤2/3, 4/≥5)	2/12/22	4/16/16
Clinical parameters		
Age (years)	49 (24-69)	59 (26-70)
Body mass index (kg/m^2^)	25.6 (20.5-36.5)	25.5 (19.1-33.6)
Serum aspartate aminotransferase (IU/l)	61 (19-152)	37 (14-132)
Serum alanine aminotransferase (IU/l)	104 (28-303)	49 (8-304)
Gamma-glutamyl transpeptidase (IU/l)	65 (17-505)	43 (9-359)
Serum albumin (g/dl)	4.1 (3.4-4.9)	4.0 (2.8-4.5)
Platelet count (×10^3^/mm^3^)	210 (117-389)	207 (111-296)
Fasting plasma glucose (mg/dl)	94 (65-142)	106 (73-278)
Uric acid (mg/dl)	6.0 (1.8-9.5)	5.9 (1.5-9.2)
Total cholesterol (mg/dl)	209 (130-290)	202 (131-270)
Triglycerides (mg/dl)	137 (62-254)	131 (54-295)
High-density lipoprotein cholesterol (mg/dl)	44 (29-85)	45 (27-73)
Low-density lipoprotein cholesterol (mg/dl)	129 (66-205)	123 (64-175)
Non high-density lipoprotein cholesterol (mg/dl)	162 (95-228)	150 (95-219)
Serum ferritin (μg/l)	265 (<10-1,472)	202 (13-1,018)
Hyaluronic acid (μg/l)	34 (8-561)	30 (0-222)
Serum miR-122 (fold change)	1.03 (0.13-7.63)	0.66 (0.03-7.65)

Data are number of patients or median (range) values

### Measurement of serum miR-122

Serum samples were obtained at least twice a year after the time of histopathological diagnosis of NAFLD. The sample was frozen at –80 °C within 4 h of collection and thawed just before analysis. Circulating microRNA was extracted from 200 μl of serum samples using the QIAGEN miRNeasy serum-plasma kit (Qiagen K.K., Tokyo) according to the instructions provided by the manufacturer. RNA was reverse transcribed using TaqMan MicroRNA Reverse Transcription kit (Life Technologies Japan, Tokyo). *Caenorhabditis elegans* miR-39 (cel-miR-39) was spiked in each sample as a control for extraction and amplification steps. Table 2 provides details of the protocol used for measurement of serum miR-122, as described previously [25]. Serum miR-122 was amplified using primers and probes provided by Applied Biosystems (Foster City, CA) by the TaqMan MicroRNA assay, according to the instructions provided by the manufacturer. The relative expression of serum miR-122 was calculated using the comparative cycle threshold (CT) method (2^-ΔΔCT^) [26, 27] with spiked cel-miR-39 as normalized internal control. The miRNA expression levels were expressed relative to the levels of serum miR-122 measured in 286 clinical samples [21]. Serum miR-122 ratio represented serum miR-122 level at second biopsy to that at first biopsy.

**Table 2:** Protocol used for analysis of serum miR-122

(A) Preparation of the RT reaction master mix			
Component	Master mix volume per 15-μL reaction^a^		
100 mM dNTPs (with dTTP)	0.15 μL		
MultiScribe^TM^ Reverse Transcriptase, 50 U/μL	1.00 μL		
10× Reverse Transcription Buffer	1.50 μL		
Rnase Inhibitor, 20 U/μL	0.19 μL		
Nuclease-free water	4.16 μL		
Total volume	7.00 μL		
(B) Performance of reverse transcription			
Use the following parameter values to program the thermal cycler:			
Step	Time	Temperature	
Hold	30 min	16 °C	
Hold	30 min	42 °C	
Hold	5 min	85 °C	
Hold	∞	4	
(C) Preparation of the qPCR reaction mix			
Pipet the following components into each tube:			
Component	Single reaction		
TaqMan^Ⓡ^ Small RNA Assay (x20)	1.00 μL		
Product from RT reaction	1.33 μL		
TaqMan^Ⓡ^ Universal PCR Master Mix II (*x*2)	10.00 μL		
Nuclease-free water	7.67 μL		
Total volume	20.00 μL		
(D) Setting up the experiment or plate documentation and running the plate			
In real-time PCR system software, create an experiment or plate document on real-time PCR system using the following parameters:			
•Run Mode: Standard			
•Sample Volume: 20 μL			
•Thermal Cycling Conditions:			
	Enzyme Activation	PCR CYCLE (40 cycles)	
Step	HOLD	Denature	Anneal/extend
Temperature	95 °C	95 °C	60 °C
Time	10 min	15 s	60 s

^a^Each 15-μL RT reaction consists of 7 μL master mix, 3 μL of 5× RT primer, and 5 μL RNA sample

### Statistical analysis

Wilcoxon test was used for comparison of paired samples. Correlation analysis was evaluated by the Spearman rank correlation test. All *p* values less than 0.05 by the two-tailed test were considered significant. Statistical analyses were performed using the SPSS software (SPSS Inc., Chicago, IL).

## Results

### Histopathological changes

Table 3 summarizes the distribution of histopathological scores at the time of first and second liver biopsies. The steatosis score indicated progression, no change, and improvement in 33.3%, 55.6%, and 11.1% of the 36 patients, respectively (Table 3), with a median change per year for the entire group of 0.000/year (range, -2.393 to 0.778/year). The ballooning score indicated progression, no change, and improvement in 13.9%, 63.9%, and 22.2% of the patients, respectively (Table 3), with a median change per year of 0.000/year (range, -2.393 to 0.178/year). Analysis of the lobular inflammation score indicated that 27.8%, 33.3%, and 38.9% of the patients showed progression, no change, and improvement, respectively (Table 3), with a median change per year of 0.000/year (range, -0.1.429 to 1.164/year). The stage scores indicated progression, no change, and improvement in 27.8%, 52.8%, and 19.4% of the patients, respectively (Table 3), with a median change per year of 0.000/year (range, -0.714 to 0.516/year).

**Table 3:** Distribution of histological scores at the time of the first and second liver biopsies

Steatosis scores						
	Scores at second biopsy					
	0	1	2	3	Total	
Scores at first biopsy						
1	0	5	3	0	8	
2	1	7	11	1	20	
3	0	3	1	4	8	
Total	1	15	15	5	36	
Ballooning scores						
	Scores at second biopsy					
	0	1	2	Total		
Scores at first biopsy						
0	0	2	0	2		
1	1	17	3	21		
2	3	4	6	13		
Total	4	23	9	36		
Lobular inflammation scores						
	Scores at second biopsy					
	0	1	2	3	Total	
Scores at first biopsy						
0	0	2	2	0	4	
1	0	8	6	0	14	
2	2	7	4	0	13	
3	0	3	2	0	5	
Total	2	20	14	0	36	
Stage scores						
	Scores at second biopsy					
	0	1	2	3	4	Total
Scores at first biopsy						
0	1	4	0	0	0	5
1	1	8	2	2	0	13
2	0	3	2	1	0	6
3	0	1	1	8	1	11
4	0	0	0	1	0	1
Total	2	16	5	12	1	36

### Association of serum miR-122 level with histopathological features

**Fig. 1:**
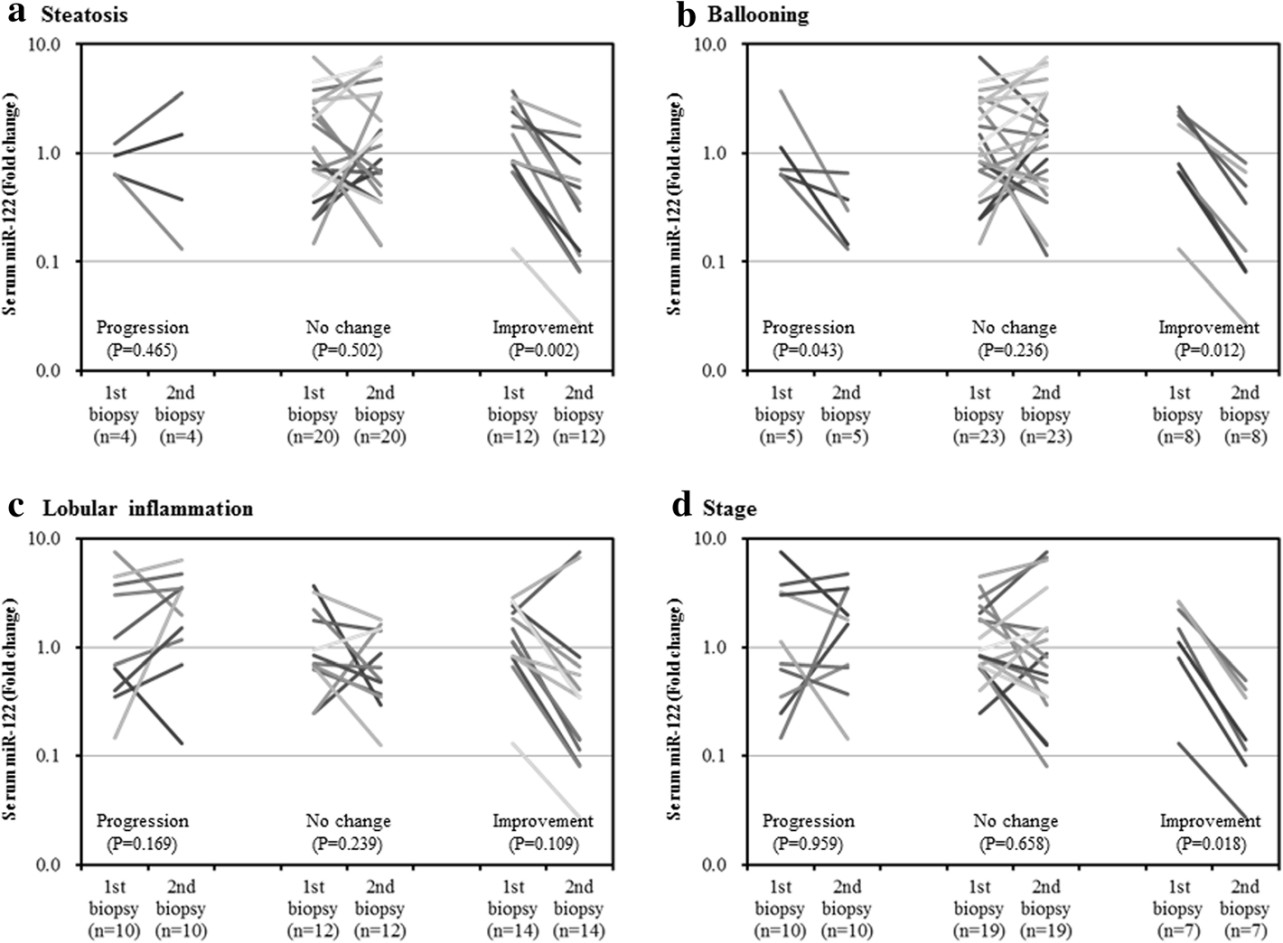
Logarithmically transformed levels of serum miR-122 at the time of first and second liver biopsies, according to histopathological features [**a** Steatosis, **b** Ballooning, **c** Lobular inflammation, and **d** Stage]. In patients with improvement of steatosis, ballooning, and stage scores, serum miR-122 levels at second biopsy were significantly lower than those at first biopsy

Figure 1 shows serum miR-122 levels at the time of the first and second liver biopsies, according to histopathological features. In 12 patients who showed improvement in steatosis scores, serum miR-122 levels were significantly lower at the second biopsy compared with first biopsy (*P* = 0.002, Fig. 1a). In 5 patients who showed progression of ballooning scores, serum miR-122 levels were significantly lower at second biopsy than at first biopsy (*P* = 0.043). In 8 patients who showed improvement of ballooning scores, serum miR-122 levels were significantly lower at second biopsy than at first biopsy (*P* = 0.012, Fig. 1b). In 7 patients who showed improvement of stage scores, serum miR-122 levels were significantly lower at second biopsy compared with at first biopsy (P = 0.018, Fig. 1d).

### Association of changes in clinical parameters with histopathological scores

**Fig. 2:**
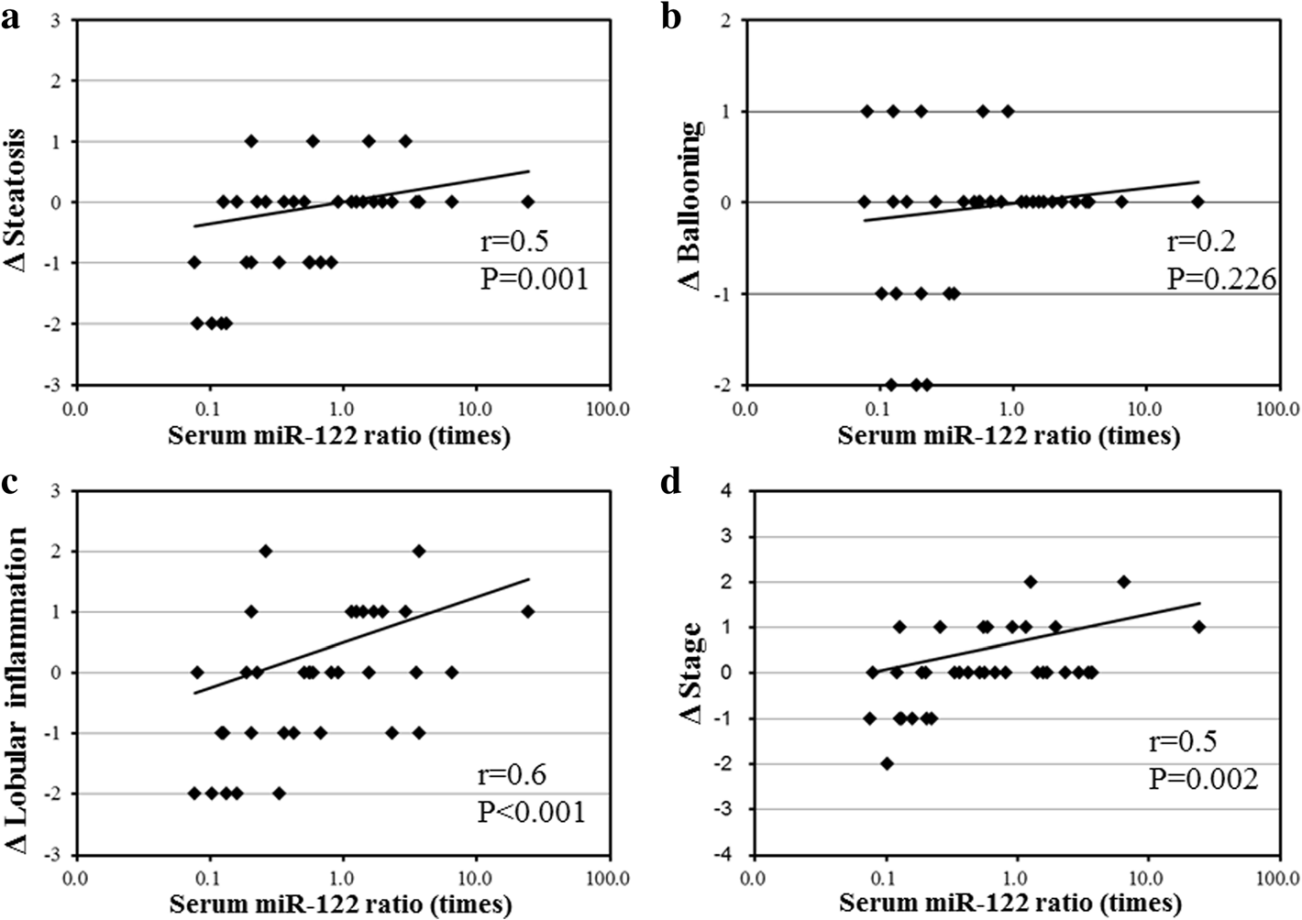
Association between changes in histopathological score [**a** ΔSteatosis, **b** ΔBallooning, **c** ΔLobular inflammation, and **d** ΔStage] with logarithmically transformed serum miR-122 ratio. Changes in histopathological scores (Δ) represented the value at second liver biopsy minus value at first liver biopsy. Serum miR-122 ratio was calculated by (ratio at serum miR-122 level at second liver biopsy/ratio at serum miR-122 level at first liver biopsy). Note the strong associations (r ≥ 0.5) between serum miR-122 ratio and changes in histopathological scores [**a** ΔSteatosis, **c** ΔLobular inflammation, and **d** ΔStage]

Table 4 shows the association of changes in clinical parameters with histopathological scores. There was significant and strong association (r ≥ 0.5) between ΔSteatosis and serum miR-122 ratio (*r* = 0.5, P = 0.001). There were also significant and strong associations between ΔLobular inflammation and ΔBMI (*r* = 0.6, *P* < 0.001), ΔAST (*r* = 0.6, *P* < 0.001), ΔALT (*r* = 0.5, P = 0.001), ΔFerritin (*r* = 0.6, *P* < 0.001), and serum miR-122 ratios (*r* = 0.6, *P* < 0.001), respectively. There were also significant and strong associations between ΔStage and ΔBMI (*r* = 0.5, *P* = 0.004), ΔAST (*r* = 0.6, *P* < 0.001), ΔHyaluronic acid (*r* = 0.5, *P* = 0.002), and serum miR-122 ratios (*r* = 0.5, *P* = 0.002). The above results pointed to a strong association between serum miR-122 ratio and changes in histopathological scores (Fig. 2).

**Table 4:** Correlation between changes in levels of clinical parameters and histological scores

	ΔSteatosis		ΔBallooning		ΔLobular inflammation		ΔStage	
	*r*	*P*	*r*	*P*	*r*	*P*	*r*	*P*
ΔAge	0.4	0.011	0.3	0.049	0.3	0.055	0.4	0.026
ΔBody mass index	0.3	0.045	0.3	0.045	0.6	<0.001	0.5	0.004
ΔSerum aspartate aminotransferase	0.4	0.018	0.3	0.126	0.6	<0.001	0.6	<0.001
ΔSerum alanine aminotransferase	0.4	0.018	0.3	0.108	0.5	0.001	0.3	0.054
ΔGamma-glutamyl transpeptidase	0.1	0.555	0.3	0.099	0.3	0.043	0.3	0.044
ΔSerum albumin	-0.1	0.748	-0.1	0.393	-0.3	0.110	-0.2	0.165
ΔPlatelet count	0.1	0.740	0.0	0.918	-0.2	0.145	-0.1	0.416
ΔFasting plasma glucose	0.0	0.913	0.2	0.390	0.3	0.102	0.3	0.100
ΔUric acid	-0.1	0.671	0.1	0.539	0.2	0.374	0.3	0.089
ΔTotal cholesterol	0.1	0.636	0.3	0.096	0.0	0.992	0.1	0.546
ΔTriglycerides	0.2	0.213	0.1	0.530	0.2	0.195	0.4	0.031
ΔHigh-density lipoprotein cholesterol	0.1	0.738	0.1	0.670	-0.3	0.056	-0.2	0.218
ΔLow-density lipoprotein cholesterol	0.0	0.942	0.2	0.248	0.0	0.855	0.0	0.849
ΔNon high-density lipoprotein cholesterol	0.1	0.675	0.3	0.109	0.1	0.597	0.1	0.385
ΔFerritin	0.3	0.048	0.3	0.063	0.6	<0.001	0.4	0.007
ΔHyaluronic acid	0.2	0.366	0.4	0.036	0.3	0.075	0.5	0.002
Serum miR-122 ratio^a^	0.5	0.001	0.2	0.226	0.6	<0.001	0.5	0.002

^a^Serum miR-122 ratio represented the ratio of serum miR-122 level at second biopsy to that at first biopsy

Changes (Δ) in levels of clinical parameters and histopathological scores were calculated by: value at second biopsy minus value at first biopsy

### Association of serum miR-122 ratio with changes in other clinical parameters

We also analyzed the relationship between serum miR-122 ratios and changes in other clinical parameters. There were significant and strong associations between serum miR-122 ratio and ΔAST (*r* = 0.7, P < 0.001), ΔALT (*r* = 0.6, *P* < 0.001), ΔGGT (*r* = 0.5, *P* = 0.003), and ΔFerritin (*r* = 0.5, *P* = 0.001).

## Discussion

There is ample evidence to suggest that many epigenetic mechanisms that are based on histone modifications, DNA methylation, microRNAs (or miRs), and ubiquitination, play a pathogenetic role in NAFLD. The miR-122 is significantly downregulated in NAFLD patients. Furthermore, inhibition of miR-122 results in downregulation of mRNA expression levels of various lipogenic genes and improvement in liver steatosis [15]. Recent studies that investigated the utility of circulating microRNA in the assessment of NAFLD found high serum levels of various microRNAs in patients with NAFLD, and reported strong association of serum miR-122 levels with histopathological disease severity [15–18]. Studies by our group also identified the lack of HCC and/or histopathological severity of NASH as independent predictors of high levels of serum miR-122 [21]. However, serum miR-122 levels tended to be low in fibrosis stage 4, and demonstrated a biphasic change with progression of fibrosis stage. Furthermore, long-term follow-up studies of HCC patients showed that serum miR-122 levels tended to decrease before the progression of fibrosis stage 4 [21]. Considered together, the above results suggest that high serum miR-122 levels could reflect potential future development of HCC, but not at present, and that low serum miR-122 levels before progression of fibrosis stage could reflect increased risk of hepatocarcinogenesis [21]. To minimize the effect of HCC on serum miR-122 levels, we excluded all patients with HCC in the present study. Using this approach, we analyzed the relation between trends in miR-122 and progression/improvement in NAFLD scores in HCC-free patients with histopathologically-confirmed NAFLD.

To our knowledge, the present observational study is the first to demonstrate the association of serum miR-122 with histological features of NAFLD. Serum miR-122 levels at the second biopsy were significantly lower than those at first biopsy in patients with improvement of histopathological scores. Furthermore, there were significantly strong associations between serum miR-122 ratio and changes in histopathological scores.

The present study has certain limitations. Our results showed that patients with improvement and progression of ballooning scores had significantly low serum miR-122 levels at second biopsy, and that serum miR-122 levels correlated with improvement in steatosis and fibrosis scores but neither with progression of steatosis nor fibrosis scores. These controversial results could be statistically due to α or β error based on the small number of patients who showed progression of steatosis. Furthermore, we recently reported that steatosis with grade of fibrosis stage 4 was significantly milder than that of fibrosis stage 0-3 (such as burned-out NASH patients with progression of fatty changes and inflammatory cell infiltration resolving in fibrosis [6]). We also showed a tendency for lower miR-122 serum levels in patients with severe fibrosis stage, especially fibrosis stage 4 [21]. In the present study, only one patient showed increase in stage scores from fibrosis stage 3 at first biopsy to stage 4 at second biopsy, and the level of serum miRNA-122 decreased from 0.71 fold change at first biopsy to 0.65 fold change at second biopsy. Further large-scale longitudinal studies using serial liver biopsies are needed to determine the complex relationship between serum miR-122 and histopathological features, including burned-out NASH.

To our knowledge, there are no studies that used serial liver biopsies to investigate changes in various clinical parameters, including serum miR-122, according to the individual components of NASH (e.g., steatosis, lobular inflammation, ballooning, and fibrosis stage). Several serial-biopsy studies have investigated the histopathological changes and predictive factors of disease progression in patients with NAFLD [28–32]. Consistent with the recently reported data [33], the present study showed significant association between changes in histopathological features and changes in levels of serum transaminase (ΔAST and ΔALT). Furthermore, there were significantly strong associations between serum miR-122 ratio and changes in other clinical parameters, including serum transaminases. One limitation of the present study, probably related to the small number of patients, is that the priority of these clinical parameters could not be determined. Further large-scale studies should be performed to identify useful surrogate markers of histopathological features.

In conclusion, longitudinal examination of serial liver biopsies showed significant association of serum miR-122 with histopathological features of NAFLD in patients free of HCC. Further studies of larger number of patients should be performed to determine the molecular mechanisms of the complex relationship between the impact of miR-122 on epigenetic risk and pathogenesis of NAFLD.

## Conclusions

Longitudinal examination of serial liver biopsies showed significant association between serum miR-122 and histopathological features of NAFLD patients free of HCC.

## Additional file

Additional file 1: Histological scores at the time of the first and second liver biopsies, and serum microRNA-122 ratio. (XLS 306 kb)

## Abbreviations

ALT: Alanine aminotransferase
AST: Aspartate aminotransferas
BMI: Body mass index
HCC: Hepatocellular carcinoma
miR-122: microRNA-122
NAFLD: Non-alcoholic fatty liver disease
NASH: Non-alcoholic steatohepatitis
